# Schizotypy but not Cannabis Use Modestly Predicts Psychotogenic Experiences: A Cross-Sectional Study Using the Oxford-Liverpool Inventory of Feelings and Experiences (O-LIFE)

**DOI:** 10.1155/2020/5961275

**Published:** 2020-10-14

**Authors:** Nicola D. Airey, Richard Hammersley, Marie Reid

**Affiliations:** ^1^NAViGO Health and Social Care CIC, 3-7 Brighowgate, Grimsby DN32 0QE, UK; ^2^Department of Psychology, University of Hull, Hull, UK

## Abstract

**Objective:**

Cannabis use predicts psychosis in longitudinal studies, but it is difficult to infer causation. Some precursor variables predict both, including childhood trauma and adversity. Additionally, some of the desired effects of cannabis use resemble the symptoms of psychosis. It would be preferable to assess psychotomimetic or “unusual” experiences that include psychotic symptoms but without assuming pathology. Finally, it is possible that similar people are prone to psychosis and drawn to cannabis use, perhaps, because they are sensitive or attracted to unusual experiences. Schizotypy provides a trait measure of proneness to unusual experiences. The study aimed to examine cross-sectionally relationships between cannabis use, schizotypy, and unusual experiences whilst controlling for current trauma symptoms.

**Method:**

A volunteer online sample (*n* = 129, 64% women, predominantly students) who had used cannabis at least once was recruited. People who reported active effects of past trauma were excluded with a brief primary care posttraumatic stress disorder screen. Participants completed the Oxford-Liverpool Inventory of Feelings and Experience, the Cognitive Failures Questionnaire, and measures of substance use and sociodemographics.

**Results:**

The majority of respondents recounted unusual experiences after cannabis use, and many of these might have been considered symptoms of psychosis if they had received medical attention. In regression analysis, the only predictor of the unusual experiences scale of O-LIFE was schizotypy (measured by the remaining subscales; 4% of variance). There were no correlations between cannabis use frequency and schizotypy or unusual experiences.

**Conclusions:**

These findings suggest that, after controlling for schizotypy and excluding people who are actively experiencing the effects of past trauma, frequency of cannabis use does not predict unusual experiences. However, individuals with schizotypal personality traits may have more unusual experiences when using cannabis.

## 1. Introduction

Whether personality or predisposition mediate the relationship between cannabis use and psychosis is an important question because psychosis is amongst the gravest possible risks of cannabis use and there were approximately 192 million cannabis users globally in 2016 [1]. In England and Wales in 2017/2018, about 6% of 16- to 59-year-olds reported cannabis use within the last year [2]. Cannabis can cause transient psychosis-like experiences [3–5], including thought disorder, paranoia, delusions, slowing of time, disturbances in visual perception, visual hallucinations, disturbances in body perception, depersonalization, and changes in mood. These are called “unusual,” “psychotogenic,” or “psychotomimetic” experiences and can resemble psychosis [6].

However, it is long known that the effects usually wear off harmlessly [3]. Indeed, some of the psychotomimetic effects are amongst the effects that users seek [7]. A few users become acutely distressed and seek medical help [8, 9]. Most frequent symptoms are paranoia with or without delusions. Treatment generally consists of reassurance and waiting for the effects to wear off. A complication in understanding the psychotomimetic effects of cannabis is that its constituents and their metabolites have long half-lives and some are stored in body fat [10]. Consequently, psychotomimetic effects experienced hours, or even days, after subjective intoxication has ended may still be caused by cannabis. Furthermore, intermittent use might sensitize the user to psychotomimetic experiences even in between bouts of use.

Indeed, of greater concern is that cannabis may have enduring psychotomimetic effects. Structural equation modelling within one large longitudinal study suggested that cannabis use plays a causal role in the development of psychotic symptoms in individuals who are genetically vulnerable [11]. A meta-analysis of longitudinal studies of cannabis use and subsequent psychotic symptoms or schizophrenia found an odds ratio of 3.90 (95% CI: 2.84 to 5.34) for the risk of psychosis outcomes for the heaviest of cannabis users in comparison with nonusers [12]. Another meta-analysis found evidence for a relationship between cannabis use and earlier age of onset of psychosis [13]. Moreover, onset of cannabis use before the age of 16 may increase the risk of psychosis fourfold by the age of 26 [14]. However, there is a problem of confounding variables [15]. Major confounds include adverse childhood experiences [16, 17] and schizotypy, which is also correlated with childhood trauma [18].

Schizotypy is a cluster of general population personality traits derived from psychosis symptomology, positive symptoms, negative symptoms, and disorganization [19, 20]. It can be linked to a pattern of general personality using five-factor personality measures [21] and its traits mirror the three-factor model of schizophrenia [22]. Schizotypy scores are moderately stable over time [23], so a high score for schizotypy is unlikely to be caused entirely by cannabis use.

Current cannabis users score more highly for schizotypal personality characteristics than nonusers and past users [24], and regular users score higher on schizotypy than less-regular users [25]. Furthermore, people with schizotypal traits tend to be more likely to experience unusual experiences following cannabis use [26]; both psychosis and schizotypy levels increase with cannabis usage in a dose-dependent manner [27] and psychotogenic symptoms after cannabis use are predicted by high schizotypy scores [26, 28].

However, some unusual experiences, including intrusions, hallucinations, and depersonalization, are also known symptoms of posttraumatic stress disorder (PTSD) and acute stress disorder [29], which may reflect positive psychotogenic symptomology. A strong relationship has been reported between schizotypal personality characteristics and trauma [30], while lifetime prevalence of cannabis use is higher amongst people with PTSD diagnoses [31]. Therefore, in interpreting findings, it is essential to be aware of these complications.

Another difficulty with the previous literature is that the measurement of “psychosis” has been inconsistent, with the use of several different questionnaires, including measures designed for clinical populations rather than the general population [19] and specially developed measures for the psychosis-like effects of cannabis [25]. Many of these measures were developed on the assumption that unusual experiences are either precursors of psychosis or undiagnosed psychotic symptoms. However, this assumption has been challenged on the basis that occasional psychotogenic experiences are not unusual in the general population and do not necessarily cause distress [32, 33]. Consequently, efforts have been devoted to developing questionnaires about such experiences that do not assume pathology but rather focus on a personality approach [19]. One leading and valid measure is the Oxford-Liverpool Inventory of Feelings and Experience (O-LIFE), which was designed for nonclinical samples [34].

The present study's aim is to examine the relationship between cannabis use and unusual experiences, controlling for schizotypy using the relevant subscale O-LIFE and excluding participants who exhibited current symptoms of PTSD [35]. It also asked participants to provide an example of an unusual experience they had had whilst using cannabis on their own, with the aim of better understanding the content of unusual experiences.

## 2. Method

### 2.1. Participants

Opportunistic anonymous volunteer sampling online was utilized by using targeted posts on social media websites (*n* = 180; 65 males and 115 females; mean age = 26.00, SD = 9.80). Inclusion required cannabis use at least once. Excluded were participants with high PTSD scores. The study aimed to recruit only participants aged 18 or older, but two participants recorded their age as 17. As the age of consent for research in the UK is 16, it was decided to retain these participants for the analysis.

### 2.2. Design

A correlational design was employed, plus a single qualitative question about an unusual experience whilst using cannabis. The variable to be predicted was the unusual experiences score. The predictor variables were age, cognitive impairment, ethnicity, education level, gender, household income, frequency of cannabis use, occupation status, other drug use, purpose of cannabis use, and schizotypal personality traits.

### 2.3. Materials

The questionnaire was presented online to allow for easy circulation, reach a larger population, and facilitate anonymity [36]. It began with an information sheet that explained the nature of the study, followed by a consent page with check box choices. It then comprised seven main sections: 1. Primary Care PTSD Screen (PC-PTSD) [37], which is using a cutoff of 3+ as the criterion for exclusion. 2. Demographics: age, gender, ethnicity, education level, occupation status, and household income based on UK tax bands [38]. 3. Cannabis use: once in my life, occasional use (a few times across my life), yearly (I use it at least once every year), monthly (I use it nearly every month), Weekly (I use it nearly every week), or frequent (I use it nearly every day). 4. Unusual experiences subscale from O-LIFE: this subscale has internal consistency (Cronbach's *α* = 0.89) [39] plus a qualitative, free text question asking for a description of one unusual experience following cannabis use. 5. Other drug use: list of 20 drugs, participants ticked each they had used, giving a score out of 20. A similar question asked about drugs used with cannabis. 6. Schizotypy using the other sections of O-LIFE; cognitive disorganization, introverted anhedonia, and impulsive nonconformity. All have internal reliability [39]—cognitive disorganization (Cronbach's *α* = 0.87), introvertive anhedonia (Cronbach's *α* = 0.82), and impulsive nonconformity (Cronbach's *α* = 0.77). 7. Cognitive Failures Questionnaire (CFQ) [40], which has strong internal reliability (Cronbach's *α* = 0.93) [41]. The complete questionnaire is available in Supplementary Materials.

### 2.4. Ethical Approval

The study was approved by the University Faculty Ethics Committee, which accords with the Declaration of Helsinki. Participation was by informed consent, and the survey was anonymous except for IP addresses that were deleted as the data were downloaded. Information and contact details for organizations, including Samaritans and Talk To Frank, were provided to participants in order for them to seek advice or help regarding what they expressed within the questionnaire and more specifically drugs and drug use. Participants were also provided with contact details for the researcher, the research supervisor, and the University's ethics committee should they need them.

## 3. Results

### 3.1. Statistical Analysis

PC-PTSD scores showed 52 participants (33 females and 19 males; age *M* = 27.25, SD = 12.52) scoring 3+ on the trauma screening measure, who were excluded. Those excluded did not differ significantly on any demographic or epidemiological variables, or on substance use. Using G^*∗*^ Power 3.1, the final sample size of 129 gave power of 0.90 to detect a small effect size (0.1) in the regression analysis reported below.

Of those included, 36% were male, and 64%, female. Mean age was 25.41 (SD = 8.47). All but 5 were white; 68% were educated to university level, 26% to college level, and only 5% less than that; 62.79% were in education (59.6% university, 3.10% college); 33% were working; and only 4% were unemployed. Household income was low for 26%, average for 59%, and high for 16%, based on the UK Tax Band criteria [38]. Thus, it was a youthful, relatively highly educated, and high-social-status sample, with women over-represented compared with typical samples of drug users.  Table 1 shows the frequency of cannabis use.

The Unusual Experiences subscale was reliable (*α* = 0.90). The mean number of types of unusual experiences was 7.2 (SD = 6.2), and the range was from 0 to 28 types of experience. The other three subscales of the O-LIFE combined to measure schizotypy was also reliable (*α* = 0.89), as was the CFQ (*α* = 0.90).

Aside from the CFQ, all other continuous variables were not normally distributed (Shapiro–Wilk *p* < 0.05); hence, nonparametric testing was undertaken.  Table 2 shows the correlations between unusual experiences, schizotypy, frequency of cannabis use, and the other variables. As shown in Table 2, unusual experiences correlated with schizotypy and cognitive impairment, while schizotypy was also correlated with cognitive impairment and age. Cannabis use was not correlated with either schizotypy or unusual experiences but was with other drug use and using other drugs simultaneously with cannabis.

Of gender, ethnicity, occupation, household income, and purpose of use, only the education level was associated with the frequency of cannabis use (Fisher's exact test, *p* = 0.010). As the data did not meet the assumption of normality, nonparametric independent-sample Kruskal–Wallis tests were undertaken, examining differences in key variables across different categories. Schizotypy (*H* [2] = 19.34, *p* < 0.001) differed across education levels. Post hoc tests indicated that there was a significant difference in schizotypy scores between individuals with university education and college education (*p* < 0.001). Schizotypy did not differ across the other variables mentioned above or by frequency of cannabis use. Unusual experiences did not differ across any category.

Prior to conducting a multiple regression analysis, the assumptions for this statistical analysis were tested. Due to the data not meeting the assumption of normal distribution, the continuous variables were transformed logarithmically before being entered into a regression analysis. Following this, the data met all assumptions for a regression analysis to avoid type I and type II errors [42]. The variables possessed nonzero variance, were lacking in autocorrelation (Durbin Watson = 2.03), and displayed linearity within scatterplots, and the residuals displayed sufficient normal distribution within the P–P plot for the model. Moreover, the analysis of collinearity statistics suggests that the assumption of non-multicollinearity was met, with all VIF scores well below 10 and all tolerance scores were above 0.2. The assumption of homoscedasticity was met as evidenced by the plot of standardized residuals versus standardized predictor values, showing no obvious signs of funnelling. Finally, Cook's distance values were all under 1, suggesting that there were not any individual cases biasing the model.

As shown in Table 3, a multiple regression analysis was conducted with unusual experiences scores as the outcome variable. Initially, control variables were entered stepwise into the first block: age, gender, ethnicity, education level, occupation status, and household income. Into the second block, the following variables were entered stepwise: O-LIFE score, CTQ score, purpose of cannabis use, number of other drugs used, and number of other drugs used with cannabis. In the final block, frequency of cannabis use was entered.

Only schizotypy was predictive of the unusual experiences score, (*F* (1, 111) = 4.53, *p* = 0.034; *R*^2^ = 0.040). No other variables added significant variance. Because schizotypy may be a risk factor for unusual experiences after cannabis, Spearman's rho was calculated correlating schizotypy and unusual experiences separately for frequent users of cannabis (at least once a week; *r*_*s*_ = 0.48) and those who used less often (*r*_*s*_ = 0.23). The correlation for the entire sample was *r*_*s*_ = 0.31. While there is no formal way of defining when one correlation is significantly larger than another, the relationship between schizotypy and unusual experiences appeared to be intensified amongst frequent users. Looking at the scatterplot, 7 participants scored >20 on unusual experiences of whom 4/7 were occasional users, 1/7 was a yearly user, and 2/7 were frequent users.

### 3.2. Unusual Experiences following Cannabis Use

Two overarching themes emerged from the qualitative responses to the question, “*Please describe one instance of an unusual experience following cannabis use in isolation/on its own*?”—symptoms of cannabis intoxication (DSM-5 criteria; [29], *n* = 51) and psychotogenic experiences (*n* = 99). Twenty-six respondents did not report any unusual experiences following cannabis use, so they were excluded, as were 3 who reported never using cannabis by itself. Another 8 responses were excluded for not providing a coherent and relevant description. Responses categorized as “cannabis intoxication” are not discussed further here, and they included intense hunger, impaired motor coordination, tachycardia, anxiety, sensation of slowed time, and euphoria.

### 3.3. Psychotogenic Experiences

**Figure 1:**
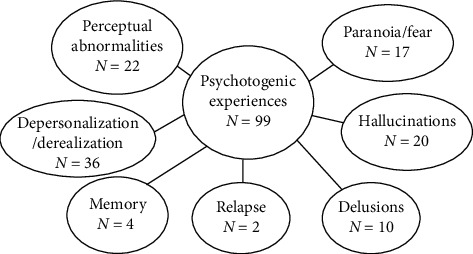
A diagram depicting the subthemes extracted from the analysis, where *N* is the number of responses.

“Psychotogenic experiences” included paranoia/fear, hallucinations, delusions, depersonalization/derealization, perceptual abnormalities, memory, and relapse. Some responses were categorized into multiple themes.  Figure 1 depicts the distribution of responses between the subthemes, which are detailed below. No participants reported that these experiences persisted beyond the period of cannabis intoxication.

#### 3.3.1. Paranoia/Fear

- Without explanation (*n* = 10): “*the feeling of dread and imminent danger with no good reason*” and “*heart palpitations and extreme terror*.”
- With explanation (*n* = 6): “*standing in my boyfriends* [sic] *kitchen a door shook and I thought it was a ghost*” and “*I thought people were outside my window, but it was a cow mooing*.”
- Persecutory delusion (*n* = 1): please see below under Section 4.3.3(b).

#### 3.3.2. Hallucinations

- Visual hallucinations (*n* = 10): “*the person I was talking to turned into a blue fuzzy number 2*” and “*seeing shadows in the corner of a room as a person, even though the details of the person were imagined beyond the details created by the shadows*.”
- Auditory hallucinations (*n* = 5): “*I could hear people in the eaves of my room*.” Another could hear a “*cat meowing to be let in,*” but there was no animal there.
- Tactile hallucinations (*n* = 2): “*felt like a hand reached up my back and grabbed my head*” and “*felt like an army of ants was crawling over my skin*.”
- (d)Olfactory and gustatory hallucinations (*n* = 2): gustatory “*water tasted like wine*” and olfactory “*overwhelmingly strong smell of freshly baked bread, even though I was in a field with no bread around*.”

#### 3.3.3. Delusions

- Grandiose delusions (*n* = 7): “*I thought I could control the clouds with my eyes*” and “*feeling more intelligent than usual, specifically* believing *I could understand human interactions in terms of atomic level movements*.”
- Persecutory delusions (*n* = 4): “*I was* 100% *sure I was about to get mugged by a guy on a motorbike*” and “*could not get the sounds of sirens out of my head. Had the constant belief that the police were going to get me.*”
- Spiritual delusions (*n* = 2): “*the presence of God*” and contact with God “*I was told to by Allah himself.*”

#### 3.3.4. Depersonalization/Derealization

- Depersonalization (*n* = 26): The most common psychotogenic experience is, “*My arms went numb and did not feel my own even when waving them*” and “*I felt like my mind had left my body*.” An example of depersonalization related to mental processes is, “*I found* it *impossible to express words and instead could only begin to spell them out letter by letter*.”
- Derealization (*n* = 10): “*almost feeling as if I'm in a bubble that surrounds me and I'm not quite in the outer world*” and “*I feel like my head goes real* [sic] *big and small really fast.*”

#### 3.3.5. Perceptual Abnormalities

- Visual (*n* = 9): “*several inanimate objects in my room (drying rack, posters) looked like figures/people*” and “*feeling as though objects (e.g., a table, a bottle on the table) were very far away even though they were right in front of me*.”
- Auditory (*n* = 4): “*I was once listening to a song and thought it was still playing for the next 2 hours when it had stopped after one play*” and “*hearing people talk to you really loud but there* [sic] *just talking normally*.”
- Strange self-perception (*n* = 7): “*I once felt alarmingly aware of my bones/skeletal structure and my muscles moving my frame*” and “*…like my head was separate to my body and felt a lot warmer than the rest of my body*.”
- Lucid dreaming (*N* = 2): “*I experience events that feel very real*” and “*I used to vividly see an image for a split second, followed by it melting away*.”

#### 3.3.6. Memory

- Memory failures (*n* = 3): “*it is hard to recall a specific incident (partly down to the effect of cannabis* [sic] *on my memory!)*” and “*I think I've said things and started conversations when I haven't*.”
- Reminiscence (*n* = 1): “*A random picture or song can transfer me back to the exact location and time it happened and make me think the same as I did back then also causes me to have the same emotions*.”

#### 3.3.7. Relapse (N = 1)

“I have had more psychotic experiences, made worse by cannabis use.”

Participants reported a wide variety of psychotogenic experiences whilst taking cannabis, which included some quite extreme delusions, hallucinations, and other distortions of perception and cognition. These experiences included many that would have met criteria for psychosis, if they had not been associated with cannabis use.

## 4. Discussion

By measuring unusual experiences rather than psychotic symptoms and excluding people who may have been currently experiencing trauma symptoms, this study found that schizotypy modestly predicted unusual experiences (4% of variance) and frequency of cannabis use did not add any additional variance. This is different from previous studies using measures of psychosis [26, 27].

In a cross-sectional study, it is impossible to tell how cannabis use may have affected schizotypy. Nonetheless, within the design of the regression analysis, schizotypy was a better predictor of unusual experiences than cannabis use. At minimum, this suggests that controlling for schizotypy eliminates the effects of cannabis use on the prevalence of unusual experiences amongst people who are not currently experiencing symptoms of trauma. None of the control variables included were predictive of unusual experiences, although schizotypy varied with educational levels and more frequent cannabis users tended to use other drugs more. There was some evidence that the relationship between schizotypy and unusual experiences was larger amongst frequent cannabis users, as reported previously [26, 27], but this relationship needs further research, ideally longitudinal. The implication is that cannabis use makes unusual experiences more likely but primarily for people already prone to unusual experiences.

It is worth noting that in this volunteer nonclinical sample, there were no significant gender differences in substance use, schizotypy, or unusual experiences. This is unusual in mental health and addiction research; often men use substances more heavily and are more prone to psychosis. It seems probable that the low entry criteria of having used cannabis at least once helped to equalize the usual gender difference, as indicated by the fact that nearly 2/3 of the sample was women. Results from this relatively light cannabis-using, predominantly female sample cannot readily be generalized to heavier cannabis-using or clinical populations.

Participants reported a wide range of psychotomimetic experiences whilst taking cannabis, and only 12% were unable to report such an experience. Many of these might have been judged to be psychotic symptoms should they have been presented to a health care professional without mentioning cannabis use. The DSM-5 acknowledges that perceptual abnormalities can occur as part of cannabis intoxication [29]. However, from what was reported here, these experiences can be quite strange and intense and there is a need to raise awareness of this amongst both users and clinicians. This is particularly important for people who score highly on schizotypy, as they may be more vulnerable to unusual experiences. With hindsight, it would have been of interest to ask also about the duration of these experiences and any distress caused, for affective content and interpretation may mediate the relationship between unusual experiences and psychosis [40, 41]. Moreover, in future research, it would be of interest to relate the nature and content of unusual experiences to schizotypy.

This study was cross-sectional, consisting of modest sample size, and a volunteer sampling method was used. Nonetheless, its findings suggest that, even if the lack of a cannabis-unusual experiences relationship after controlling for schizotypy does not replicate, studies of cannabis and psychotomimetic experiences (or psychotic symptoms) should consider schizotypy as well. The established relationship between cannabis use and psychotic symptoms is complicated by personality, by any enduring effects of past trauma, and by how participants choose to manage their cannabis use based on what experiences they have had with it. These factors need to be considered carefully in future research.

## Figures and Tables

**Table 1:** Frequency of cannabis use within the sample of 129 participants.

Frequency	*N*	%
Once in their lifetime	10	7.75
Occasional use across their lifetime	41	31.78
At least once a year	12	9.30
At least once a month	26	20.16
At least once a week	16	12.40
Frequently (nearly every day)	24	18.60

**Table 2:** Spearman's rho correlation coefficients between the ordinal variables within the study.

	Schizotypy	Cannabis use frequency	Cognitive impairment	Other drugs	Other drugs with cannabis	Age
Unusual experiences	^*∗∗*^0.31	0.03	^*∗∗*^0.23	−0.06	0.15	−0.12
Schizotypy		−0.08	^*∗∗*^0.55	−0.12	0.03	^*∗∗*^−0.39
Cannabis use frequency			−0.02	^*∗∗*^0.44	^*∗∗*^0.57	−0.14

^*∗∗*^ *p* < 0.01 level (two-tailed).

**Table 3:** Summary of the stepwise regression analysis for variables predicting unusual experiences.

	*B*	*β*	*p*
Model 1			
Constant	0.37 (−0.81, 1.55)		0.534
Schizotypy	0.40 (0.03, 0.76)	0.20	0.034

Excluded variables		*T*	*p*
Frequency of cannabis use		1.13	0.262
Age		−0.16	0.876
Gender		1.85	0.067
Ethnicity		1.19	0.238
Education level		0.56	0.577
Occupation status		−0.15	0.878
Household income		−0.85	0.399
Purpose of cannabis use		0.43	0.672
Number of other drugs used		0.43	0.521
Number of other drugs used with cannabis		0.64	0.530
Cognitive impairment		−0.12	0.901

95% confidence intervals are reported in parentheses. Statistics for the excluded variables can also be seen within the table.

## Data Availability

The full data set is available from the corresponding author on request.
